# A new species of *Microsphecodes* from Jamaica (Hymenoptera, Halictidae)

**DOI:** 10.3897/zookeys.111.1597

**Published:** 2011-06-22

**Authors:** Michael S. Engel

**Affiliations:** 1 Division of Entomology, Natural History Museum, and Department of Ecology & Evolutionary Biology, 1501 Crestline Drive – Suite 140, University of Kansas, Lawrence, Kansas 66049-2811, USA

**Keywords:** Hymenoptera, Apoidea, Anthophila, Halictinae, Halictini, Sphecodina, new species, taxonomy

## Abstract

A new species of the cleptoparasitic bee genus *Microsphecodes* Eickwort and Stage (Halictinae: Halictini) is described and figured from a male and female collected in Jamaica. *Microsphecodes xaymacensis* Engel, **sp. n.**, is distinguished from its congeners on the basis of integumental coloration and sculpturing, and form of the male pygidial plate and genitalia.

## Introduction

The West Indian sphecodine fauna is, much like the general halictine fauna of the region, poorly documented. Relatively few species have been described and recorded from the West Indies (Table 1) and for nearly all, the biology remains to be characterized [*e.g*., Raw 1985 (mention of unidentified species); Eickwort 1988]. The West Indian halictid fauna is considered mostly by Eickwort (1988), Snelling (2005), (Engel (2001a, 2006a, 2006b), (Genaro (2001, 2006, 2007, 2008), and Genaro and Franz (2008), with minor additions by Smith-Pardo (2009) and Engel (2011), but revisions and critical biological investigations are needed greatly. Hosts remain unknown for all of the described West Indian sphecodines.

Herein I provide a brief contribution to this fauna by describing a new species of *Microsphecodes* from Jamaica, the first representative of this genus from the island.

**Table 1.** Checklist of world species of *Microsphecodes*, and other described Caribbean sphecodines1 (from Engel 2006a, 2006b, 2006c).

**Genus** *Microsphecodes* Eickwort & Stage		
	–Continental	
	*Microsphecodes kathleenae* (Eickwort)	Costa Rica, Colombia
	*Microsphecodes russeiclypeatus* (Sakagami & Moure)	Brazil
	*Microsphecodes trichommus* Michener	Colombia
	*Microsphecodes truncaticaudus* Michener	Colombia
	–Caribbean	
	*Microsphecodes dominicanus* (Stage)	Dominica
	*Microsphecodes kittensis* Engel	St. Kitts
	*Microsphecodes solitarius* (Ashmead)	St. Vincent2
	*Microsphecodes thoracicus* (Ashmead)	St. Vincent
	*Microsphecodes xaymacensis* sp. n.	Jamaica
**Genus** *Nesosphecodes* Engel		
	*Nesosphecodes anthracinus* Engel	Puerto Rico
	*Nesosphecodes cubicola* Engel	Cuba
	*Nesosphecodes halictophagus* Engel	Dominican Republic
**Genus** *Sphecodes* Latreille		
	*Sphecodes genaroi* Engel	Cuba
	*Sphecodes nigritus* Ashmead	St. Vincent
	*Sphecodes tainoi* Engel	Cuba

1 Sphecodines as a whole are quite diverse and the monophyly of *Sphecodes* is questionable. Once phylogenetic studies are completed then the resurrection of former entities such as *Drepanium*, *Proteraner*, and *Sphecodium* should be considered over a retrograde classification lumping all into *Sphecodes* as has been advocated.

2 The apparent bias in diversity towards St. Vincent (with three sphecodines recorded) is artificial and simply reflects that this is one of the few islands, along with Grenada, for which there is a significant historical monograph (Ashmead 1900). These islands, which are of average size for the Lesser Antilles, do not harbor a greater diversity and once the sphecodines of Dominica, Barbados, Saint Lucia, Curaçao, Guadeloupe, and Martinique are thoroughly surveyed the diversity will undoubtedly grow.

## Material and methods

Material is deposited in the Snow Entomological Collection, Division of Entomology, University of Kansas Natural History Museum, Lawrence (SEMC), and the Florida State Collection of Arthropods, Gainesville (FSCA). Morphological terminology follows that of Engel (2001b) and Michener (2007), and the format that of Engel (2006a). Measurements were made using an ocular micrometer on an Olympus SZX12 stereomicroscope, while photographs were prepared with a Nikon D1x digital camera attached to an Infinity K-2 long-distance microscope lens.

## Systematics

### Genus *Microsphecodes* Eickwort and Stage, 1972

#### Microsphecodes xaymacensis

Engel sp. n.

urn:lsid:zoobank.org:act:3048683A-BF8D-4474-A2F3-BD47C2AB3126

http://species-id.net/wiki/Microsphecodes_xaymacensis

Figs 1 [Fig F2] –12

##### Holotype.

 ♂, Jamaica: Saint Andrew Parrish, Hard war Gap, 2–3-viii-1985 [2–3 August 1985], C.B. & H.V. Weems, G.B. Edwards (FSCA).

##### Paratype.

 ♀, Jamaica: Saint Andrew Parrish, Hard war Gap, 2–3-viii-1985 [2–3 August 1985], C.B. & H.V. Weems, G.B. Edwards (SEMC).

##### Diagnosis.

 The new species can be readily distinguished from its congeners by the structure of the male genitalia (Figs 6–8) and the combination of the broad male pygidial plate bordered by elongate simple or apically-branched setae (Fig. 4), hyaline wings (Fig. 5), and the rugoso-striate basal area of the propodeum not enclosed by carinae (Figs 3, 12) (the latter generally typical for West Indian species: *vide* Eickwort and Stage 1972). In addition, the coloration of the new species deviates from other West Indian *Microsphecodes* in the entirely ferruginous or orange-testaceous mesosoma [in *Microsphecodes solitarius* (Ashmead) the entire mesosoma is black except the pronotum and mesosternum are testaceous; in *Microsphecodes dominicanus* (Stage) the entire mesosomal dorsum is black, the pleura are fuscous, and the venter testaceous; in *Microsphecodes thoracicus* (Ashmead) the mesoscutum and pleura are testaceous while the mesoscutellum, metanotum, and propodeum are darkly infuscate; and in *Microsphecodes kittensis* Engel the entire mesosoma is yellow to yellow-testaceous with the mesoscutellum and metanotum black].

##### Description.

###### Male:

Total body length 4.85 mm; forewing length 4.1 mm. Head broader than long (width 1.33 mm, length 1.04 mm as measured from clypeal apex to vertex in frontal aspect) (Fig. 2). Frontal line carinate just between antennal toruli to point above upper tangent of toruli equivalent to about torulus diameter, becoming an impressed line from that point onward. Mandibular base meeting lower border of compound eye. Inner margin of compound eye slightly concave just above level of antennal toruli. Gena narrower than compound eye in profile. Scape length 0.42 mm; first flagellomere about as long as second flagellomere. Intertegular distance 0.89 mm. Forewing venation as in figure 5; hind wing with six distal hamuli arranged in a single series. Pygidial plate well delimited, wide, broadly rounded at apex, with slightly depressed shining surface and carinate rim (Fig. 4). Male genitalia as in figures 6–8.

Integument generally shining. Clypeus imbricate with shallow, contiguous punctures; remainder of head distinctly punctate, punctures on lower part of face nearly contiguous, becoming more widely spaced toward upper frons and vertex, separated by 0.25–1.5 times a puncture width, integument between punctures smooth and shining except on lower face finely imbricate, punctures weaker on vertex and sparser around ocelli; postgena faintly imbricate and impunctate. Pronotum with sparsely-scattered, minute punctures, integument between punctures imbricate. Mesoscutum imbricate with punctures separated by 1–2.5 times a puncture width, punctures shallower, fainter, and sparser around median line and along anterior and lateral sections; tegula impunctate and exceedingly faintly imbricate; mesoscutellum sculptured as on mesoscutum except punctures fainter and separated by 2–3 times a puncture width. Metanotum imbricate. Pleura smooth to faintly imbricate, with sparse minute punctures. Basal area of propodeum with strong, rugulose striae radiating from basal margin (Fig. 3), integument between striae finely imbricate; lateral and posterior surfaces of propodeum imbricate with scattered, faint, coarse punctures. Metasomal terga and sterna faintly imbricate except first metasomal tergum smooth.

**Figures 1–5.**
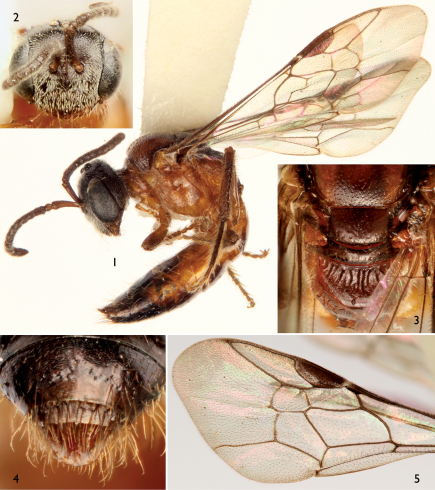
Photomicrographs of male holotype of *Microsphecodes xaymacensis* Engel, sp. n. **1** Lateral habitus **2** Facial aspect **3** Dorsal view of posterior mesosoma, highlighting propodeum, metanotum, mesoscutellum and posterior third of mesoscutum **4** Pygidial plate **5** Detail of forewing venation.

Mandible, labrum, and labiomaxillary complex ferruginous; remainder of head nearly black or dark brown; antenna dark brown (Figs 1–2). Mesosoma largely ferruginous (Fig. 1) except darker on median and lateral portions of mesoscutum and entirety of mesoscutellum, metanotum, and dorsal surface of propodeum (Fig. 3). Wing veins brown; wing membrane largely hyaline. Legs ferruginous except meso- and metatibiae and meso- and metatarsi brown. Metasoma largely ferruginous except dark brown on more apical terga and sterna; pygidial plate ferruginous (Fig. 4).

Pubescence relatively sparse, white except somewhat yellow on pleura, legs, and metasoma. Setae generally simple and erect, some with minute branches; face with moderately-dense, appressed, short, plumose setae on lower face and clypeus (Fig. 2).

###### Female:

As described for the male except in usual gender differences as well as the following: Total body length 4.80 mm; forewing length 4.2 mm. Head broader than long (width 1.41 mm, length 1.04 mm). Mandible elongate, without dentition, about as long as compound eye (Figs 9–10). Frontal line carinate just between antennal toruli to point above upper tangent of toruli equivalent to twice torulus diameter, becoming a faintly impressed line from that point onward (Fig. 10). Gena only slightly narrower than compound eye in profile (Fig. 9). Scape length 0.52 mm; first flagellomere slightly shorter than second flagellomere. Intertegular distance 0.89 mm. Inner metatibial spur simple.

Mandible and labiomaxillary complex orange testaceous; labrum, clypeus and face dark reddish brown blending to nearly black on vertex (Figs 10–12); gena dark reddish brown; scape and pedicel orange testaceous; flagellum dark brown; mesosoma orange testaceous except more yellowish on pronotal dorsal surface and propodeal dorsal surface (Figs 11–12); legs orange testaceous except dark reddish brown to ferruginous on meso- and metatibiae and meso- and metatarsi; metasoma orange testaceous blending to ferruginous and to dark brown by third tergum, remaining terga largely ferruginous, with dark brown apical portions.

Mesoscutal punctures more well defined posteriorly and separated by 1–1.5 times a puncture width, otherwise as in male with punctures shallower and fainter anteriorly and more widely spaced.

Setae on legs white and on apical portions of metasoma fuscous.

##### Etymology.

 The specific epithet is based on the indigenous Arawakan-speaking Taíno islanders’ name for Jamaica, Xaymaca, and meaning “Land of Springs”.

**Figures 6–8. F2:**
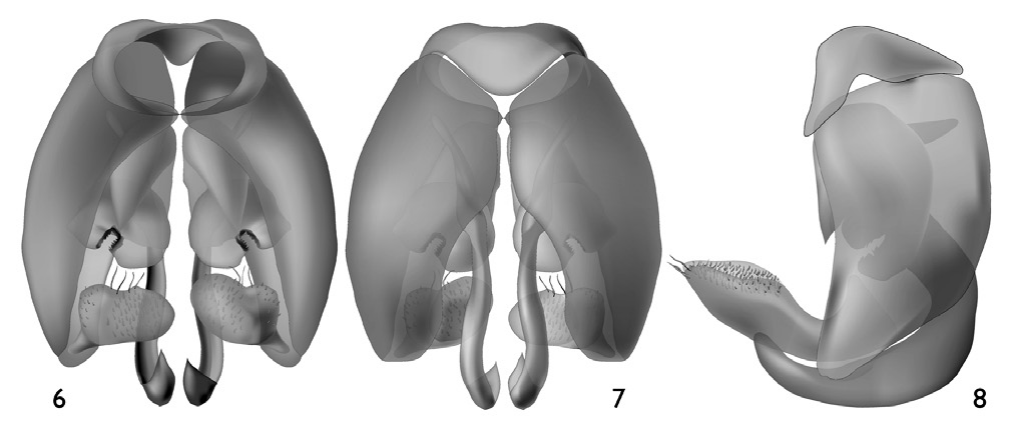
Male genitalia of *Microsphecodes xaymacensis* Engel, sp. n. **6** Ventral aspect **7** Dorsal aspect **8** Lateral aspect.

**Figures 9–12.**
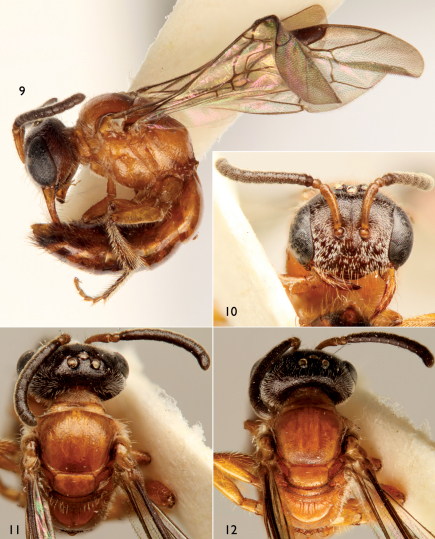
Photomicrographs of female paratype of *Microsphecodes xaymacensis* Engel, sp. n. **9** Lateral habitus **10** Facial aspect **11** Dorsal view of head and mesosoma highlighting head and mesoscutum **12** Dorsal view of head and mesosoma highlighting mesoscutellum, metanotum, and propodeum.

## Supplementary Material

XML Treatment for Microsphecodes xaymacensis

## References

[B1] Ashmead WH (1900) Report upon the aculeate Hymenoptera of the islands of St. Vincent and Grenada, with additions to the parasitic Hymenoptera and a list of the described Hymenoptera of the West Indies. Transactions of the Royal Entomological Society of London 48 (2):207-367. 10.1111/j.1365-2311.1900.tb02379.x

[B2] Eickwort GC (1988) Distribution patterns and biology of West Indian sweat bees (Hymenoptera: Halictidae). In: Liebherr JK (Ed) Zoogeography of Caribbean Insects. Cornell University Press, Ithaca, 231–253 [total volume ix+[i]+285 pp.]

[B3] Eickwort GC Stage GI (1972) A new subgenus of Neotropical *Sphecodes* cleptoparasitic upon Dialictus (Hymenoptera: Halictidae, Halictinae). Journal of the Kansas Entomological Society 45 (4):500-515.

[B4] Engel MS (2001a) Three new *Habralictellus* bee species from the Caribbean (Hymenoptera: Halictidae). Solenodon 1:33-37.

[B5] Engel MS (2001b) A monograph of the Baltic amber bees and evolution of the Apoidea (Hymenoptera). Bulletin of the American Museum of Natural History 259:1-192. doi:10.1206/0003-0090(2001)259;lt0001:AMOTBA;gt2.0.CO;2

[B6] Engel MS (2006a) A new species of *Microsphecodes* from St. Kitts (West Indies) (Hymenoptera: Halictidae). Mitteilungen des Internationalen Entomologischen Vereins 31(1–2): 51–54, +1 pl.

[B7] Engel MS (2006b) A new genus of cleptoparasitic bees from the West Indies (Hymenoptera: Halictidae). Acta Zoologica Cracoviensia 49B(1–2): 1–8.

[B8] Engel MS (2006c) The *Sphecodes* of Cuba (Hymenoptera: Halictidae). Acta Zoologica Cracoviensia 49B(1–2): 73–78.

[B9] Engel MS (2011) A new species of *Dialictus* from Sombrero Island, Anguilla (Hymenoptera, Halictidae). ZooKeys 86:61-68. 10.3897/zookeys.86.909 PMC3082990 21594093

[B10] Genaro JA (2001) Tres especies nuevas del género *Lasioglossum* (*Dialictus*), grupo *Habralictellus* para Cuba (Hymenoptera: Halictidae). Solenodon 1:38-44.

[B11] Genaro JA (2006) A history of systematic studies of the bees of Cuba (Insecta: Hymenoptera, Anthophila). Zootaxa 1195:39-60.

[B12] Genaro JA (2007) Las abejas (Hymenoptera: Apoidea: Anthophila) de la Hispaniola, Antillas. Boletín Sociedad Entomológica Aragonesa 40:247-254.

[B13] Genaro JA (2008) Origins, composition and distribution of the bees of Cuba (Hymenoptera: Apoidea: Anthophila). Insecta Mundi 52:1-16.

[B14] Genaro JA Franz NM (2008) The bees of Greater Puerto Rico (Hymenoptera: Apoidea: Anthophila). Insecta Mundi 40:1-24.

[B15] Michener CD (2007) The Bees of the World [2nd Edition]. Johns Hopkins University Press, Baltimore, MD, xvi+[i]+953 pp., +20 pls.

[B16] Raw A (1985) The ecology of Jamaican bees (Hymenoptera). Revista Brasileira de Entomologia 29 (1):1-16.

[B17] Smith-Pardo AH (2009) A new species of *Habralictus* (Hymenoptera, Halictidae) from the Island of Grenada (Lesser Antilles) with comments on the insular species of the genus. ZooKeys 27:51-58. 10.3897/zookeys.27.265

[B18] Snelling RR (2005) Wasps, ants, and bees: aculeate Hymenoptera. In: Lazell JD (Ed) Island: Fact and Theory in Nature. University of California Press, Berkeley, 283–296 [total volume xx+382 pp.]

